# Tanaidaceans (Crustacea) from the Central Pacific Manganese Nodule Province. I. The genera *Collettea*, *Robustochelia* and *Tumidochelia*

**DOI:** 10.3897/zookeys.87.784

**Published:** 2011-03-24

**Authors:** Kim Larsen

**Affiliations:** CIMAR/CIIMAR (Centro Interdisciplinar de Investigação Marinha e Ambiental), LMCEE (Laboratory for Marine Community Ecology and Evolution), Rua dos Bragas 289, 4050–123 Porto, Portugal

**Keywords:** Tanaidacea, Tanaidomorpha, *Collettea*, *Robustochelia*, *Tumidochelia*, Manganese nodule province

## Abstract

Three new species of are described from the manganese nodule province between the Clarion and the Clipperton Fracture Zone of the equatorial North Pacific Ocean, and collected during the Nodinaut expedition on board the r/v *l´Atalante* in the summer of 2004. The new species belongs to three genera as: *Collettea (Collettea longisetosa)*, *Robustochelia (Robustochelia pacifica)*, and *Tumidochelia* (*Tumidochelia tuberculata*). A key to the genus *Tumidochelia* is presented and the validity of the genera *Robustochelia* and *Collettea* is discussed.

## Introduction

The aim of this paper is to present the first part of the taxonomic results from a survey of the Central Pacific Manganese Nodule Province between the Clarion and the Clipperton Fracture Zone of the equatorial North Pacific Ocean. This region has, due to the rich manganese deposits, been a tempting area for mineral exploration. This fact has made the area one of the best surveyed deep-sea regions in the Pacific. For the Tanaidacea alone this has resulted in a couple of publications (Wilson 1987; Larsen 1999b; 2000) and more are under way (Gurreo-Kommritz pers comm.; Larsen research in progress).

The phylogeny and systematics of the Tanaidacea is still very much unresolved despite recent advances. Large groups of genera are still left without family affiliation and the diagnoses of many families are incomplete or even contradictory. Unfortunately the reduced general morphology of tanaidaceans, the conservative morphology of many otherwise not closely related taxa, and the huge diversity of especially the deep-sea taxa, makes it hard to resolve the systematic confusion without genetic data (Larsen and Froufe 2010).

Any collection in remote deep-sea habitats will reveal a significant proportion of undescribed species relative to known species (Larsen and Froufe 2010): the ratio of unknown/known species in deep-sea sampling is estimated to be about 80/20 and even higher at remote or previously unstudied locations (Larsen unpublished data). An additional problem is the fact that most species are represented by one specimen only further hampering systematic treatment.

## Material and methods

Samples were taken during the Nodinaut expedition (17 May – 28 June 2004) from on board of the research vessel *l´Atalante* using an Usnel Box corer and the submersible *Nautile* with a spade corer (Carottier a lame). The material was sieved over a 0.5 mm sieve and fixed in 4% buffered formalin and stored in 70% alcohol afterwards.

Dissections were made in glycerine using chemically sharpened tungsten wire needles. Body length was measured from the tip of the cephalothorax to the apex of the pleotelson. The terminology in the descriptions is based on Larsen (2003). Types are deposited in the Museum National d’Histoire Naturelle Paris (MNHN).

## Taxonomy

**Order Tanaidacea Dana, 1849**

**Suborder Tanaidomorpha Sieg, 1980**

**Superfamily Paratanoidea Lang, 1949**

### Family Colletteidae Larsen & Wilson, 2002

#### Genus Collettea

(G.O. Sars, 1882)

##### Type species.

*Strongylura cylindrata* (G.O. Sars, 1882).

##### Diagnosis.

(Modified after Larsen 2005) Female. Body almost completely cylindrical. Carapace longer than wide. Eye-lobes small but present, without visual elements. Pleon and pleotelson not fused. Pleotelson mostly longer than last three free pleonites combined, terminating in dorsal plate covering uropods. Antennule with 4–5 (minute terminal article) articles. Antenna with 6–7 (fusion line) articles. Mandibles with small, but not pointed crushing area. Maxillule setal number varying, some setae may be bifurcate or setulose. Maxillipedal endites without inner distal setae. Maxilliped palp article 2 without multifurcate seta. Chelipeds attached via lateral sclerite. Pereopods slender, with coxae, and with dactylus and unguis not fused to hook. Pleopods absent in females. Uropods biramous; endopod with one or two articles; exopod uniarticulated.

Male. Similar to female. Functional mouthparts retained in adult. Antennule thicker than in female. Pleopods present, most often with simple setae.

##### Remarks.

*Collettea* is a genus well represented in all major oceans from less than 100 meters (G.O. Sars 1896) to abyssal depths exceeding 5000 meter (Kudinova-Pasternak 1973; 1983). It is recognized by elongated pleotelson. The genus currently consists of 18 species including the one described herein.

##### Gender.

Feminine.

##### Species currently assigned to Collettea:

*Collettea alicjae* Błażewicz-Paszkowycz & Larsen, 2005; *Collettea antarctica* (Vanhöffen, 1914); *Collettea arnaudi* (Shiino, 1978); *Collettea cylindrata* (G.O. Sars, 1882); *Collettea cylindratoides* Larsen, 2000, *Collettea elongata* Larsen, 2002, *Collettea humbolti* Larsen, 2000, *Collettea larviformis* (Kudinova-Pasternak, 1973); *Collettea lilliputa* Błażewicz-Paszkowycz & Larsen, 2005; *Collettea longipedia* Kudinova-Pasternak, 1986; *Collettea longipleona* Błażewicz-Paszkowycz & Larsen, 2005; *Collettea longisetosa* sp. n.; *Collettea minima* (Hansen, 1913); *Collettea pegmata* Bamber, 2000; *Collettea rotundicauda* Kudinova-Pasternak, 1983; *Collettea subtilis* Kudinova-Pasternak, 1981; *Collettea vermiformis* (Lang, 1971); *Collettea wilsoni* Larsen, 1999a

##### Collettea longisetosa sp. n.

urn:lsid:zoobank.org:act:E2231109-FF50-4099-A046-A3FAC467981D

Figs 1 [Fig F2] 3 

###### Material examined.

Holotype male (MNHN-Ta1029) SUB-CStation MAC 3 #43, 07/06–2004, 13°42.3490'N, 131°29.9940'W, depth > 4000 m.

###### Diagnosis male.

Body 19 times longer than broad. Antenna article 4 without fusion line. Maxilliped endite with small inner process. Cheliped propodus with setulose inner ridge; fixed finger with multiple prominent sharply pointed processes. Cheliped dactylus not longer than fixed finger. Heavy armament with spiniform setae on all pereopods. Pleopods uniramous and strongly elongated; endopod with four long (longer than antennule) plumose setae. Uropods long (0.3 times pleotelson length), endopod biarticulated.

###### Etymology.

Named to reflect the long setae of the pleopods.

###### Description.

Adult male, 2.9 mm (body and appendages of holotype).

**Figure 1.**
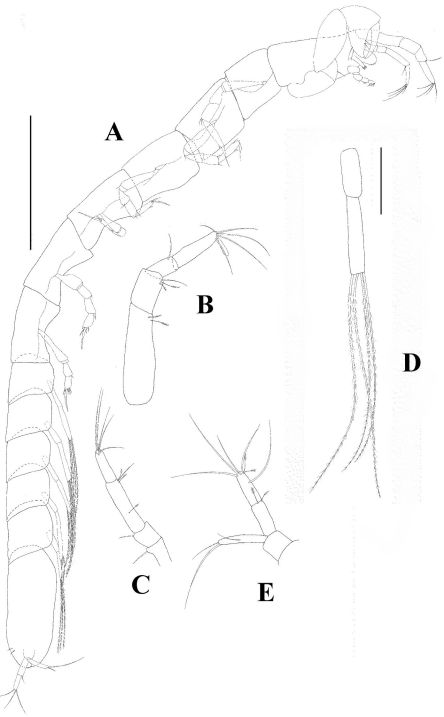
*Collettea longisetosa* sp. n., male. Holotype **A** lateral view, scale bar = 0.5 mm **B** antennule **C** antenna **D** pleopod **E** uropod. Scale bars = 0.1 mm.

*Body* (Fig. 1A) strongly elongated, 19 times as long as broad.

*Cephalothorax* longer than combined length of pereonite 1 and 2.

*Pereon*. Pereonites 1 and 2 wider than long. Pereonites 3–6 longer than wide.

*Pleon* long (about 75% as long as rest of body). All pleonites subequal, bearing long uniramous setae.

*Pleotelson* as long as combined length of three pleonites.

*Antennule* (Fig. 1B) with four articles, terminal article fused with article 4 and creating a dorsal projection. As long as cephalothorax. Article 1 marginally shorter than rest of antennule, with two setulated distal setae. Article 2 shorter than article 4, with one simple and two setulated distal setae. Article 3 half as long as article 4, with one simple distal seta. Article 4 half as long as article 1, with five simple distal setae and one short but wide aesthetasc.

*Antenna* (Fig. 1C) length 0.8 times length of antennule. Article 1 naked and fused to the cephalon. Article 2 elongated and widening distally, with one robust dorsodistal seta. Article 3 shorter than article 2, with one robust dorsodistal seta. Article 4 longer than other articles, with one medial setulose seta but without recognizable fusion line, with one simple and two setulated distal setae. Article 5 longer than article 2, with one simple distal seta. Article 6 minute with four distal setae.

**Figure 2. F2:**
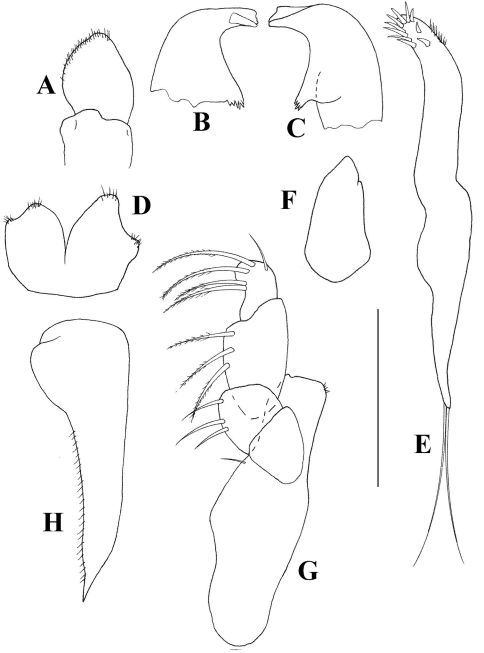
*Collettea longisetosa* sp. n., male. Holotype **A** labrum **B** left mandible **C** right mandible **D** labium **E** maxillule **F** maxilla **G** Maxilliped **H** epignath. Scale bars = 0.1 mm.

*Mouthparts*: Labrum (Fig. 2A) acorn-shaped, with finely setulose apex. Mandibular molarwith very wide basis, tapering into a small crushing area with five-six terminal spines. Left mandible (Fig. 2B) incisor with two indiscrete distal denticles, *lacinia mobilis* prominent but simple. Right mandible (Fig. 2C) incisor wide, with two distal denticles. Labium (Fig. 2D) with medially setulose projections and finely setulose apex.Maxillule (Figure 2E) endite with ten spiniform distal setae and dorsal setulation. Palp longer than endite, with two long terminal setae. Maxilla (Fig. 2F) acorn. Maxilliped (Fig. 2G) basis with one small simple seta. Endites with small inner distal process and fine outer setulation. Palp article 1 smooth. Article 2 with three outer setae. Article 3 with three finely setose setae. Article 4 with four finely setose and one simple distal setae. Epignath (Fig. 2H) longer than maxillule endite, with finely setose inner margin but naked apex.

**Figure 3.**
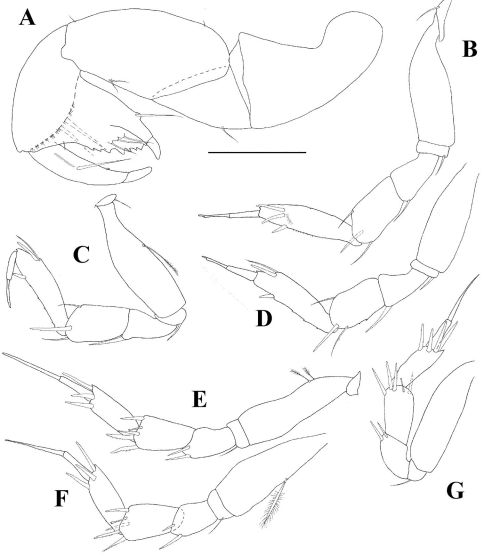
*Collettea longisetosa* sp. n., male. Holotype **A** cheliped **B** pereopod 1 **C** pereopod 2 **D** pereopod 3 **E** Pereopod 4 **F** pereopod 5 **G** pereopod 6. Scale bar = 0.1 mm.

*Cheliped* (Fig. 3A) basis divided unequally by sclerite, marginally shorter than carpus. Merus triangular with one ventral seta. Carpus longer than propodus including fixed finger, with two small simple ventromedial setae and one small simple seta dorsoproximal and dorsodistal. Propodus with setulose inner ridge, with two robust setae, with one simple outer seta at dactylus insertion; fixed finger with multiple prominent sharply pointy processes and three inner seta and one ventral seta arising from a prominent tubercle; with ventral ridge terminating in unguis. Dactylus with conspicuous inner seta, row of setules and one inner spine.

*Pereopod 1* (Fig. 3B) coxa with one simple seta. Basis fairly robust, shorter than three succeeding articles together, naked. Ischium with one robust simple seta. Merus shorter than carpus, widening distally, with one simple ventrodistal seta. Carpus shorter than dactylus, with one spiniform and two simple distal setae. Propodus more than half as long as basis, with three spiniform subdistal setae, row of scales and dorsal spine. Dactylus and unguis combined shorter than propodus. Unguis with subdistal ventral expansion at exit of spinning gland.

*Pereopod 2* (Fig. 3C) as pereopod 1 except: basis with medioventral setulated seta, merus with one spiniform distal seta, propodus with two spiniform and one simple subdistal setae and dorsal spine; ventral margin with row of spines.

*Pereopod 3* (Fig. 3D) as pereopod 2 except basis naked.

*Pereopod 4* (Fig. 3E) coxa indistinct. Basis with two dorsoproximal setulated setae. Ischium with regular simple ventral seta. Merus with two spiniform ventrodistal setae. Carpus with four spiniform (two of which are finely serrated) and one bone-shaped distal setae. Propodus as long as carpus, with three spiniform distal setae. Dactylus and unguis combined longer than propodus, not fused, and without spinning gland exit.

*Pereopod 5* (Fig. 3F) as pereopod 4 except basis with one medioventral setulated seta.

*Pereopod 6* (Fig. 3G) as pereopod 4 except basis naked. Propodus with four spiniform distal setae.

*Pleopods* (Fig. 1D) uniramous and strongly elongated. Basal article shorter than endite, naked. Endopod with four long (longer than antennule) sparsely plumose setae.

*Uropod* (Fig. 1E) basal article shorter than exopod, naked. Endopod with two articles subequal length articles; article 1 with two setulated distal setae; article 2 with one long subdistal, four long simple and two setulated distal setae. Exopod longer than endopod article 1, with one medial and two unequally length distal setae.

###### Remarks.

It is hard to compare *Collettea longisetosa* and other *Collettea* species given that it is a male and most other species are described from females only. The sexual dimorphism of *Collettea*, know from the species, which have described male (*Collettea minima* (Larsen, 2000) *Collettea elongate* (Larsen, 2002) and *Collettea lilliputa* (Błażewicz-Paszkowycz & Larsen, 2005) indicate only minor differences between the sexes in the antennule and absence of pleopods in the females. The remarkable uniramous pleopods and their equally remarkable setae of this species separate it from any other male described.

A similar armament of the cheliped fixed finger with its strong inner serration and transverse row of inner setae/setules is also seen in *Collettea subtilis* Kudinova-Pasternak, 1981 and *Collettea rotundicauda* Kudinova-Pasternak, 1983, but none of these species have the ventral fixed finger setae arising from a tubercle or the prominent dactyli seta. Furthermore in the case of *Collettea subtilis* the pereonites 2–5 are of similar size and in *Collettea rotundicauda* the antennule is much more robust (despite being a female, which usually have slender antennules than male), a shorter antennule article 4, and a shorter pleon. The powerful setae of the pereopods is also recorded from *Collettea longipleona* Błażewicz-Paszkowycz & Larsen, 2005, but this species have shorter and uniarticulated uropod. Another important feature of this species is the ischial setae on the pereopods 1–3, which are much more robust than usually seen in tanaidacean ischial setae. The crushing area of the mandibular molar, while not exactly pointed, is unusual small compared to what is seen in other species of *Collettea.* However, the long pleotelson still indicate that the species belong to *Collettea*, or to a new genus if/when *Collettea* is spilt into several genera. The only other genus that show some similarities, like the elongated body, cheliped structure and species with variable mandibular molar width, is *Filitanais* Kudinova-Pasternak, 1973 but the pleon is too short and the pleotelson too long in the new species to fit the diagnosis of that genus.

##### Collettea sp.

Fig. 4 

###### Material examined.

Manca II SUB-CStation SUB-C # 1, 05/06–2004, 14°01.7913'N, 130°08.1755'W, depth 4987 m.

###### Description.

Manca II

*Body* (Fig. 3A) length 1.9 mm. Ten times as long as broad. Cephalothorax shorter than combined length of pereonite 1 and 2.

*Pereon*. Pereonites 1, 5, and 6 wider than long. Pereonite 2–4 longer than wide.

*Pleon* relatively short, about half as long as rest of body. All pleonites subequal but progressively deeper towards pleotelson.

*Pleotelson* longer than combined length of three pleonites, deeper than pleonites.

*Pereopod 4* (Fig. 3B) dactylus with clear ventral spine at unguis insertion.

**Figure 4.**
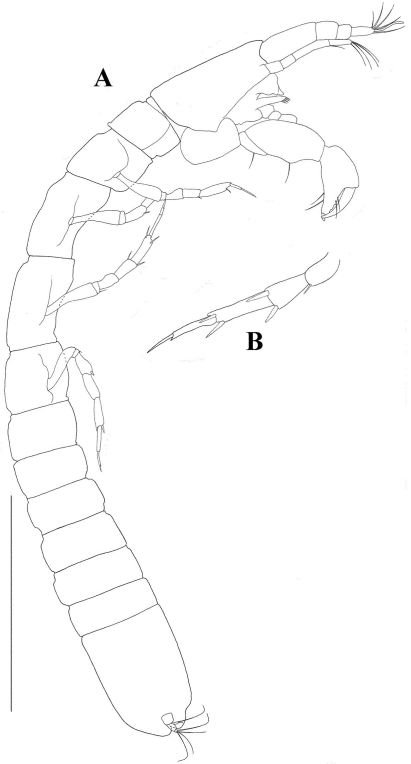
*Collettea* sp. **A** lateral view, scale bar = 0.5 mm **B** tip of pereopod 4, scale bar = 0.5 mm.

###### Remarks.

As this species was only recorded from one specimen of stage manca II it is not justifiable to erect a new species. The short uropods indicate however that it is not conspecific with *Collettea longisetosa*. This manca could be conspecific with *Collettea* sp. (Larsen 2000) which was collected in the same ocean basin (09°37.41'N, 151°45.00'W), however, since that species was not dissected, no further comparisons can be made. Several other species of *Collettea* share the ventral dactylus spine on pereopods 4–6 (*Collettea alicjae* Błażewicz-Paszkowycz & Larsen, 2005; *Collettea humbolti* Larsen, 2000; *Collettea longipleona* Błażewicz-Paszkowycz & Larsen 2005) and the species recorded here and thus this character is not good for species identification.

#### Genus Tumidochelia

Knight, Larsen & Heard, 2003

##### Type species.

*Tumidochelia randyi* Knight, Larsen & Heard, 2003.

##### Diagnosis.

See Larsen and Shimomura (2007)

##### Gender.

Feminine.

##### Remarks.

The genus *Tumidochelia* is a much rarer genus than *Collettea*, but is fairly well defined by the combination of a large cheliped carpus shield and a biramous uropod with an additional spiniform distal process on inner margin of the basal article. The genus is encountered both in fairly shallow water around 100 meters (G.O. Sars 1896**)** and at abyssal depths below 5000 meters (this study). It currently consists of five species including the one described herein.

The mouthparts of this genus are remarkably small relative to the size of the whole animal. When compared to a specimen of the genus *Tanais* or most other shallow-water tanaidomorphans of a similar body size, the differences in the size of the mouthparts are several hundred percent.

##### Species currently assigned to this genus.

*Tumidochelia dentifera* (G.O. Sars, 1896); *Tumidochelia knighti* Larsen and Shimomura, 2007; *Tumidochelia randyi* Knight, Larsen & Heard, 2003; *Tumidochelia tuberculata* sp. n.; *Tumidochelia uncinata* (Hansen, 1913).

##### Tumidochelia tuberculata sp. n.

urn:lsid:zoobank.org:act:5A3B3689-9B38-42E9-AC76-CEAF7ECE6077

Figs 5 [Fig F6] 7 

###### Material examined.

**Holotype,** non-ovigerous female (MNHN-Ta1031), Station CAROT-0 # 15, 04/06–2004, 14°02.8654'N, 130°05.3508'W, depth 5044 m.

###### Diagnosis.

Female. Pereonites 2–4 longer than wide. Pleotelson longer than combined length of four pleonites. Antenna with six articles (+ fusion line), article 4 longer than other articles, with clear fusion line. Cheliped propodus with large paired dorsal tubercles near dactylus insertion. Pereopod 1 merus and carpus with long (longer than length of merus) robust setae.

Male. Unknown.

###### Etymology.

The species is named after the diagnostic character of the tubercles on the cheliped propodus.

###### Description.

Female(body and appendages of holotype).

**Figure 5.**
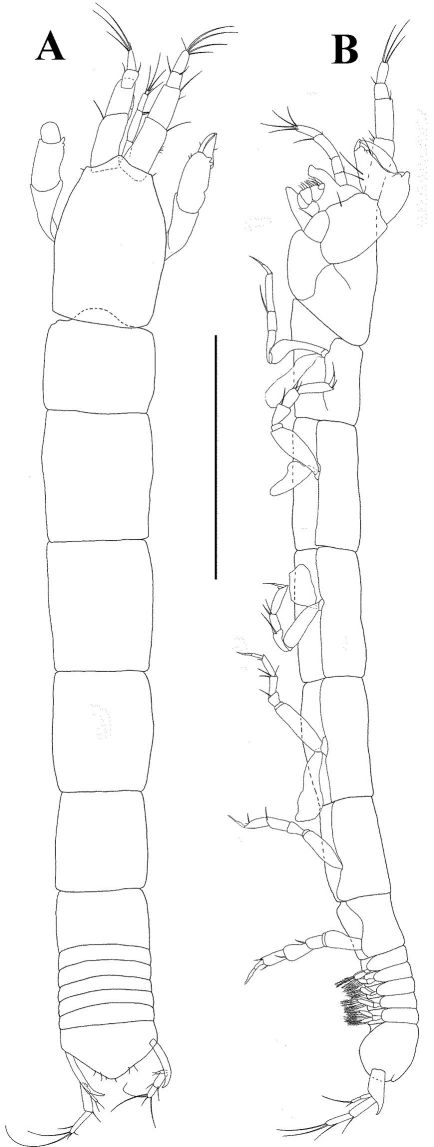
*Tumidochelia tuberculata* sp. n. Holotype **A** dorsal view **B** lateral view Scale bar 0.5 mm.

*Body* (Fig. 5A, B). Body length 2.0 mm. Subcylindrical, elongate, approximately 8.5 times longer than wide. Lateral edges almost completely straight.

*Cephalothorax* longer than wide (l/w 1.5). Eyes and eye-lobes absent.

*Pereon.* Pereonites 1 and 6 wider than long. Other pereonites longer than wide, pereonites 3 longest.

*Pleonites* all wider than long, subequal, bearing pleopods.

*Pleotelson* longer than combined length of four pleonites.

**Figure 6. F6:**
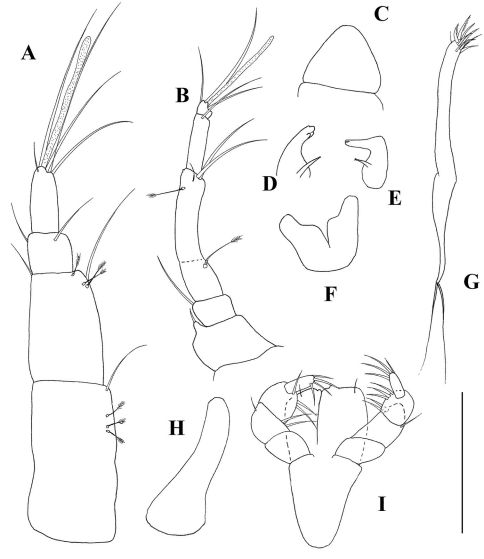
*Tumidochelia tuberculata* sp. n. Holotype **A** antennule **B** antenna **C** labrum **D** right mandible **E** left mandible **F** labium **G** maxillule **H** maxilla **I** Maxilliped. Scale bar 0.1 mm.

*Antennule* (Fig. 6A) with four articles. Stout at base- tapering distally, almost as long as carapace. Article 1 with one simple distal seta and three subdistal setulated setae; article 2 approximately 0.80 times as long as article 1, with two simple and three setulated distal setae; article 3 approximately 0.3 times length of article 2, with two simple distal setae; article 4 approximately twice length of article 3, with four long and one short simple distal setae and one prominent aesthetasc.

*Antenna* (Fig. 6B) approximately 0.7 times as long as antennule. Article 1 naked and fused to cephalon. Article 2 wider than other articles, with dorsodistal process and one stout dorsodistal seta. Article 3 band-shaped, with one dorsodistal seta, Article 4 longer than other articles, with clear fusion line, with one medial (distal on first article component) setulose seta, with two long and one short simple setae distally and with one subdistal setulated setae. Article 5 with one simple distal seta. Article 6 approximately 0.25 length of article 5, with three simple distal setae and one aesthetasc.

*Mouthparts*:Relatively small compared to body size (mandibular body less than 0.1 mm, see remarks). Labrum (Fig. 6C) apex pointed, apparently naked. Mandibular molar tapering. Left mandible (Fig. 6D) incisor with three distal denticles; *lacinia mobilis* blunt with two denticles. Right mandible (Fig. 6E) incisor with two denticles. Labium (Fig. 6F) with one pair of lobes, without setules or process. Maxillule (Fig. 6G) endite with nine terminal spiniform setae of which two are serrated; palp with two terminal setae. Maxilla (Fig. 6H) narrow and long (as long as maxillule endite), featureless. Maxilliped (Fig. 6I) endites with blunt distal process and small outer seta, fairly wide (almost as wide as basis) and long (reaching palp article 4). Basis fused. Palp article 1 naked; article 2 with two inner setae and one outer seta; article 3 with three inner setae; article 4 with four setae. Epignath not recovered.

**Figure 7.**
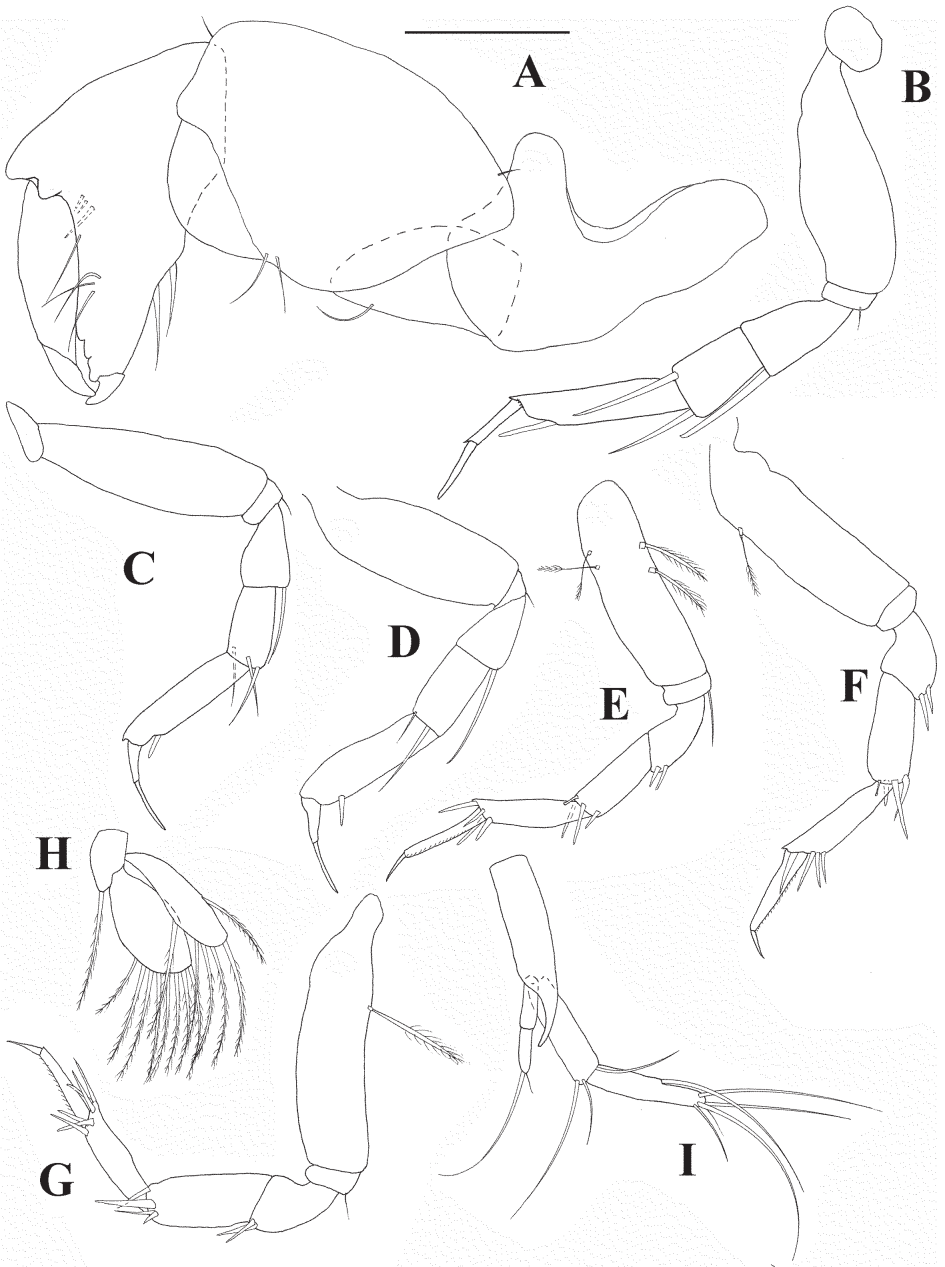
*Tumidochelia tuberculata* sp. n. Holotype **A** cheliped **B** pereopod 1 **C** pereopod 2 **D** pereopod 3 **E** Pereopod 4 **F** pereopod 5 **G** pereopod 6 **H** pleopod **I** uropod. Scale bar 0.1 mm.

*Cheliped* (Fig. 7A) attached to cephalothorax by a large sclerite. Basis naked, narrow in posterior part, approximately as long as carpus. Merus with one ventral seta. Carpus widening distally, with two small dorsal setae and two simple ventromedial setae, ventrodistal part inflated into a large carpal shield, extending distally past propodus articulation. Propodus as long as basis, with two simple (thick) ventral setae mid-length and three (one longer than the other two) inner setae proximal to dactylus insertion, with paired dorsal crest next to dactylus insertion. Fixed finger with three inner setae and three blunt denticles. Dactylus as long as fixed finger

*Pereopod 1* (Fig. 7B) longer than other pereopods. Coxa naked. Basis robust without seta. Ischium with one simple distal seta; merus widening distally, longer than carpus, with one ventrodistal, long (longer than merus) bayonet-shaped setae. Carpus rectangular, half as long as propodus, with two long (longer than merus) bayonet-shaped distal setae. Propodus elongate, longer than merus, with one spiniform ventro-subdistal seta and dorsal spine. Dactylus and unguis combined shorter than propodus, dactylus with distal spine at unguis insertion. Unguis as long as dactylus.

*Pereopod 2* (Fig. 7C) as pereopod 1 except it is smaller; carpus with three long setae; dactylus without spine.

*Pereopod 3* (Fig. 7D) as pereopod 2 except: carpus with two setae.

*Pereopod 4* (Fig. 7E) coxa absent. Basis with two medial setulated setae on both margins (the ventral ones much the larger). Merus with two short spiniform ventral setae. Carpus with three spiniform ventral setae and one bone-shaped seta. Propodus with four spiniform distal setae. Dactylus (including unguis) as long as propodus, with ventral serration; unguis less than 0.3 times as long as dactylus.

*Pereopod 5* (Fig. 7F) as pereopod 4 except: basis with only one dorsal setulated seta. Ischium apparently naked.

*Pereopod 6* (Fig. 7G) as pereopod 4 except: basis with only one ventral setulated seta. Propodus, with six distal setae.

*Pleopods* (Fig. 7H). Basal article with one plumose seta. Endopod rectangular, with numerous simple distal setae. Exopod rectangular, with one robust proximal seta, and numerous simple distal setae.

*Uropod* (Fig. 7I) biramous, basal article naked, with dorsomedial spiniform process. Endopod with two subequal articles; article 1 with three simple distal setae; article 2 with one long simple subdistal seta, three long and two short simple distal setae. Exopod reaching beyond midlenght of first endopod article, with two subequal articles; article 1 naked, article 2 with two simple but unequally length distal setae.

###### Remarks.

*Tumidochelia tuberculata* can be separated from *Tumidochelia uncinata* by the cheliped propodus having paired dorsal crest next to dactylus insertion; from *Tumidochelia dentifera* by the pleotelson being longer than last three pleonites combined; from *Tumidochelia randyi* by the pereonite 2 being longer than other pereonites; from the only other Pacific species, *Tumidochelia knighti*, by the straight lateral margins (with no segment indentations), the longer pleotelson, the antenular fusion line, the cheliped propodus tubercles, and a longer uropodal exopod.

###### Key to the species of Tumidochelia, females

**Table:** 

1	Pleotelson longer than last three pleonites	2
–	Pleotelson shorter than last three pleonites	4
2	Pereonites of subequal length and width. Antenna article 4 without fusion line	*Tumidochelia randyi*
–	Pereonite 2 longer than other pereonites. Antenna article 4 with clear fusion line	3
3	Cheliped propodus with paired dorsal crest next to dactylus insertion	*Tumidochelia tuberculata*
–	Cheliped propodus without paired dorsal crest next to dactylus insertion	*Tumidochelia uncinata*
4	Antenna article 4 with clear fusion line. Uropodal exopod almost reaching the end of first endopod article	*Tumidochelia dentifera*
–	Antenna article 4 without fusion line. Uropodal exopod barely reaching beyond midlenght of first endopod article	*Tumidochelia knighti*

### Family Incertae sedis

#### Genus Robustochelia

Kudinova-Pasternak, 1983

##### Type species.

*Leptognathia robusta* (Kudinova-Pasternak, 1970)

##### Diagnosis.

(Modified after Józwiak and Błażewicz-Paszkowycz 2007) Female. Antennule with four visible articles and one minute terminal article (often not visible in the compound scope as with *Collettea* and many other tanaidomorphans). Antenna with six articles, articles 2 and 3 with robust dorsodistal setae. Mandibular molar pointed. Labium consists of one pair of lobes with minute distal setulation, usually with medial processes (e.i. ´lobes´ sensu Józwiak and Błażewicz-Paszkowycz 2007). Maxillipedal palp robust; endites not fused, with distal setae but without distal denticles, narrower than basis. Chelipeds attached by ventral sidepiece, carpus and propodus massive, dactylus and fixed finger shorter than rest of propodus, and heavily chitinized. Pereopods 1–3 with coxa, dactylus and unguis combined shorter than, or as long as, propodus, unguis longer than dactylus. Pereopods 4–6 without coxa, dactylus and unguis not fused to a claw. Pleopods present. Uropodal exopod uniarticulated, endopod consisting of one (with pseudoarticulation) or two articles.

Male:Similar to female, but antennule articles 1–3 much wider than in female.

##### Gender.

Feminine.

##### Species currently assigned to this genus.

*Robustochelia angusticephala* Kudinova-Pasternak, 1986; *Robustochelia longa* Kudinova-Pasternak, 1983; *Robustochelia pacifica* sp. n.; *Robustochelia robusta* (Kudinova-Pasternak, 1970); *Robustochelia virilis* Józwiak and Błażewicz-Paszkowycz, 2007.

##### Remarks.

The genus *Robustochelia* is a rare, and exclusively deep-sea, genus (Larsen 2005; Józwiak and Błażewicz-Paszkowycz 2007). It is primarily recognized by the heavy cheliped with a calcified keel on the cheliped fixed finger but despite the recent clarifications by Józwiak and Błażewicz-Paszkowycz (2007) it i still poorly defined and poorly studied genus, due to the rarity of both species and specimens. The genus currently consists of five species including the one described herein. Apart from the heavy cheliped, the defining characters of *Robustochelia* are the combination of a ‘general leptognatid´ morphology: consisting of an antennule with four plus one minute articles, a pointed mandibular molar, and a uniarticulated exopod. However the calcified cheliped keel is found in species of other genera (*Leptognathioides*, *Siphonolabium*, *Monstrotanais*, and *Robustochelia*[?] *solida*) and the combination of the other ´general leptoganthid´ characters all present in several other deep-sea genera (*Caudalonga*, *Filitanais*, *Forcipatia*, *Leptognathia*, *Leptognathiella*, *Stenotanais*). No other synapomorphic characters are present and several of the species of *Robustochelia* are only incompletely described. Unfortunately it is not unusual for tanaid systematists to have to rely on a character combination as this for genus diagnosis, but the homoplastic nature of all the characters in *Robustochelia* indicates that it is probably not monophyletic. Further studies, particularly genetic studies, needs to verify the status of this genus (Larsen and Froufe in progress).

The labial medial processes or ´lobes´ *sensu* Józwiak and Błażewicz-Paszkowycz (2007) are in this authors opinion note ´lobes´ in the true sence as seen in *Leptochelia* or *Tanais* but thin membranous lateral extensions).

##### Robustochelia pacifica sp. n.

urn:lsid:zoobank.org:act:64828C04-B759-4400-A3DD-B35DC486A0D1

Figs 8 [Fig F9] 10 

###### Material examined.

**Holotype** manca II (MNHN-Ta1030) SUB-CStation CAROT-0 # 18, 05/06–2004, 14°02.6665'N, 130°05.9948'W, 5045 m.

###### Diagnosis.

Manca II. Carapace longer than the lengths of pereonites 1 and 2 combined. Pereonites 2–5 longer than wide. Pleotelson longer than combined length of three pleonites. Mandibular molar tapering. Maxillipedal endites with medial setae. Cheliped propodus and proximal part of fixed finger with calcified keel; fixed finger with clear tubercle distal from the keel; dactylus with dorsoproximal seta. Pereopods 1–3 propodus setae only reaching unguis insertion. Uropods as long as pleotelson.

Male. Unknown.

###### Etymology.

Named after the Pacific Ocean.

###### Description.

Manca II (body and appendages of holotype).

**Figure 8.**
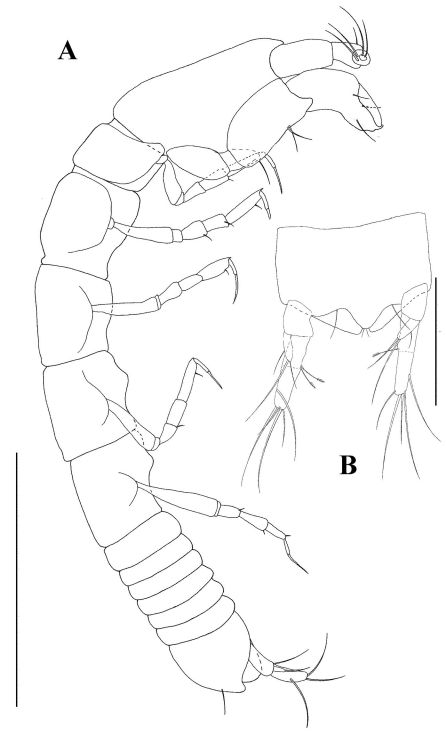
*Robustochelia pacifica* sp. n. Holotype **A** lateral view **B** Pleotelson and uropods. Scale bars 0.5 mm.

*Body* (Fig. 8A) length 1.7 mm. About ten times as long as wide (owing to the curved nature of the holotype, body morphometric calculations are based on the lateral view).

*Cephalothorax* more than 1.5 times as long as wide. Shorter than the lengths of pereonites 1 and 2 combined. Eye-lobes absent.

*Pereon* lateral margins almost straight. Pereonite 1 and 6 wider than long. Pereonites 2–5 longer than wide.

*Pleon* short (including pleotelson about 0.25 times total body length).

*Pleotelson* longer than the lengths of three free pleonites combined, apex blunt and covered by dorsal plate.

**Figure 9. F9:**
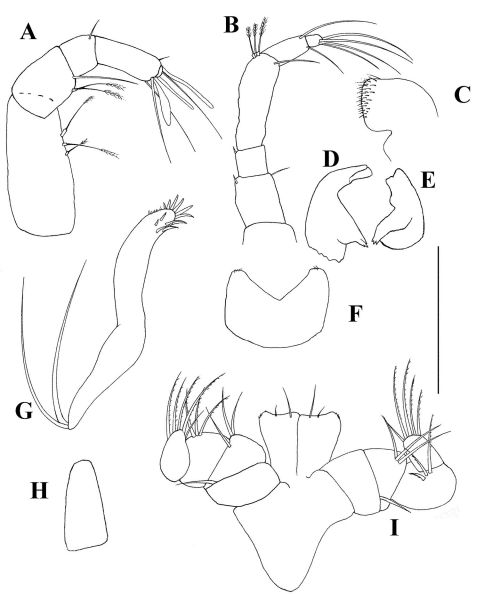
*Robustochelia pacifica* sp. n. Holotype **A** antennule **B** antenna **C** labrum **D** left mandible **E** right mandible **F** labium **G** maxillule **H** maxilla **I** Maxilliped. Scale bar 0.1 mm.

*Antennule* (Fig. 9A) 0.67 times as long as carapace, with four plus one minute terminal articles. Article 1 shorter than rest of antennule, with three setulose setae medially. Article 2 longer than article 3, with two setulated and one simple distal setae. Article 3 shorter than article 4, with one simple distal seta. Article 4 longer than article 2, with two long and one short simple distal setae, with one very wide distal aesthetasc. Article 5 minute, with two aesthestascs and one seta.

*Antenna* (Fig. 9B) with six articles. Almost as long as antennule. Article 1 wider than long, naked, not significantly wider than other articles. Article 2 longer than article 3, with one simple dorsal and ventral setae. Article 3 longer than article 5, with one simple dorsal seta. Article 4 longer than other articles, with three setulose and one simple distal setae. Article 5 as long as article 2, with two simple distal setae. Article 6 minute, with four distal setae.

*Mouthparts*: Labrum (Fig. 9C) finely setose, with flat apex. Mandibular molar tapering, ending in a few small spines. Left mandible (Fig. 9D) *lacinia mobilis* longer and broader than incisor; incisor blunt. Right mandible (Fig. 9E) incisor with medial depression. Labium (Fig. 9F) as wide as maxillipedal endites, outer corners with only a few setules. Maxillule (Fig. 9G) endite with 12 spiniform distal setae; palp with two terminal setae. Maxilla (Fig. 9H) elongated and featureless. Maxilliped (Fig. 9I) endites without denticles but with two distal setae, the medial one longest; palp article 1 with one outer seta, article 2 with three inner setae, article 3 with three setae on inner margin, article 4 with five setae. Epignath not recovered.

**Figure 10.**
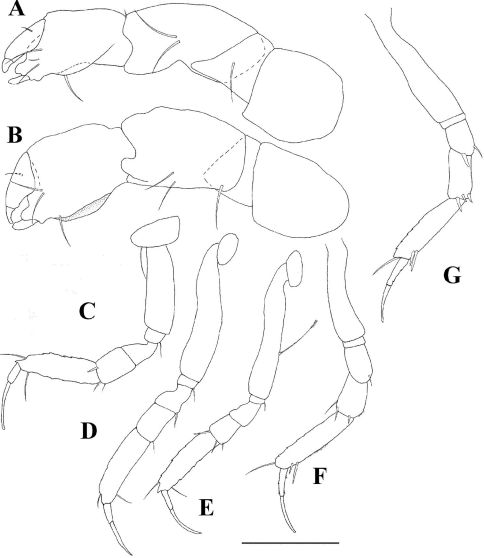
*Robustochelia pacifica* sp. n. Holotype **A** left cheliped, inner view **B** right cheliped, outer view **C** pereopod 1 **D** pereopod 2 **E** pereopod 3 **F** Pereopod 4 **G** pereopod 5 **H** pereopod 6 **I** pleopod. Scale bar = 0.1 mm.

*Cheliped* (Fig. 10A, B) basis shorter than carpus, naked. Merus prominent with one simple ventral seta. Carpus appears twisted in relation to propodus, shorter than propodus including fixed finger, with two simple ventral setae and prominent distal process. Propodus massive, with one seta at dactylus insertion and ventral calcified keel. Fixed finger with keel only on proximal part, but with a clear tubercle distally from the keel, with one ventral seta and two on inner margin, and with one large distal process. Dactylus as long as fixed finger, with small medial process and small dorsoproximal seta.

*Pereopod 1* (Fig. 10C) coxa with one simple seta. Basis longer than the three succeeding articles combined. Ischium with one ventral seta. Merus as long as carpus, widening distally, naked. Carpus shorter than 0.5 times propodus, with two spiniform and one simple distal setae. Propodus with spinnules and one distal seta on both margins and dorsal spine. Dactylus and unguis combined shorter than propodus, not fused, dactylus shorter than unguis.

*Pereopod 2* (Fig. 10D) as pereopod 1 except: coxa apparently without seta. Merus with ventral seta. Carpus with simple ventral seta.

*Pereopod 3* (Fig. 10E) as pereopod 1 except: basis with ventromedial setulose seta.

*Pereopod 4* (Fig. 10F) with no visible coxa. Basis about as long as the three succeeding articles combined, naked. Ischium naked. Merus with one simple ventral seta. Carpus with two small spiniform and one simple distal setae. Propodus longer than carpus, with two spiniform ventral and one simple dorsal setae. Dactylus and unguis combined shorter than propodus, not fused, with spinnules on both margins. Unguis still longer than dactylus, but shorter than in pereopod 1–3.

*Pereopod 5* (Fig. 10G) as pereopod 4 except merus with two spiniform setae and carpus with three.

*Pereopod 6* absent in manca II.

*Pleopods* absent in manca II.

*Uropod* (Fig. 8B) longer than pleotelson. Basal article naked and about as long exopod. Endopod with weak traces of a medial pseudo-articulation, with two medial setulose setae, one subdistal seta and four distal setae. Exopod less than half of endopod length, with two distal setae of unequal length.

###### Remarks.

Bacause the studied specimen represent manca stageit is difficult to compare it with the other species of the genus. The carapace being longer than combined length of pereonites 1 and 2 and the weak armament of the pereopods (particularly the carpus) differentiate *Robustochelia pacifica* sp. n. from *Robustochelia longa*. As for *Robustochelia angusticephala***,** this species is only incompletely described and nothing is known about the mouthparts so comparison with this species is difficult. However, the weaker fixed finger of *Robustochelia pacifica* and the shape of the cephalothorax indicates that these are not conspecific.*Robustochelia pacifica* most closely resemble *Robustochelia robusta*, but differs in the stout antennule (particularly article 4) and antenna, but also the more rectangular shape of the cephalothorax, the pereonites 2–5 being longer than wide, the large tubercle distally on the fixed finger, and the much longer pleotelson. *Robustochelia pacifica* differs from *Robustochelia virilis* by the pereonite 4 and 5 being longer than wide and by the pereopods 1–3 propodus setae not reaching longer than unguis insertion.

The species *Robustochelia solida* Larsen, 2005 was removed from the genus by Józwiak and Błażewicz-Paszkowycz 2007, based primary on the present of the present of a broad molar. This removal is undoubtly correct and thus clearly illustrate that the heavy cheliped is not a sufficient character for defining the genus *Robustochelia*.

## Supplementary Material

XML Treatment for Collettea

XML Treatment for Tumidochelia

XML Treatment for Robustochelia
